# Data on OCT and fundus images for the detection of glaucoma

**DOI:** 10.1016/j.dib.2020.105342

**Published:** 2020-02-28

**Authors:** Hina Raja, M. Usman Akram, Sajid Gul Khawaja, Muhammad Arslan, Aneeqa Ramzan, Noman Nazir

**Affiliations:** a Dept. of Computer Engineering, National University of Sciences and Technology Islamabad, Pakistan; b Dept. of Mechanical Engineering, National University of Sciences and Technology Islamabad, Pakistan; c Department of Electrical Engineering, National University of Technology (NUTech), Islamabad, Pakistan; d Armed Forces Institute of Ophthalmology, Rawalpindi, Pakistan

**Keywords:** Fundus images, OCT images, Optic nerve head (ONH), Cup to disc ratio (CDR) and glaucoma

## Abstract

This paper presents the data set of Optic coherence tomography (OCT) and fundus Images of human eye. The OCT machine TOPCON'S 3D OCT-1000 camera is employed to acquire the images. The dataset is comprised of 50 images which includes control and glaucomatous images. For each OCT Image there is a corresponding fundus Image with annotation. Cup to disc ratio (CDR) values annotated by glaucoma specialists through fundus Images are provided in excel file. OCT images are optic nerve head (ONH) centred. Manually annotation is performed for the delineation of the Inner Limiting Membrane (ILM) Layer and Retinal pigmented epithelium (RPE) layer with the help of ophthalmologist. The data is valuable for the development of automated algorithm for glaucoma diagnosis.

**Table:** Specifications Table

Subject	Ophthalmology
Specific subject area	Human retina, Glaucoma
Type of data	Images Excel file
How data were acquired	Images are acquired using TOPCON'S 3D OCT-1000 system.
Data format	1. JPG (.jpg) raw Images, Optic coherence tomography (OCT) Images are resolution of 951 × 456. 2. JPG (.jpg) Manually Annotated Images 3. Annotated values in EXCEL file (.xlsx)
Parameters for data collection	Optic Nerve Head (ONH), Cup to Disc Ratio (CDR), Retinal Layers
Description of data collection	OCT and fundus Images of human eye. We provide the OCT image and its Fundus image along with annotation done by glaucoma specialists.
Data source location	The data under investigation is obtained from Armed Forces Institute of Ophthalmology (AFIO). Rawalpindi, Pakistan 33.5962° N, 73.0450° E
Data accessibility	Repository name: Mendeley Data Data identification number: doi.org/10.17632/2rnnz5nz74.2 Direct URL to data: https://data.mendeley.com/datasets/2rnnz5nz74/2
Related research article	[1]. T. Khalil, M. U. Akram, H. Raja, A. Jameel and I. Basit, “Detection of Glaucoma Using Cup to Disc Ratio From Spectral Domain Optical Coherence Tomography Images,” *IEEE Access,* vol. 6, pp. 4560–2576, 2018.

**Table:** 

**Value of the Data** • The data is valuable for the development and improvement of automated algorithm for glaucoma detection. • The provided data is expedient for the analysis of retinal layers especially optic nerve head (ONH) region. • The data can be useful for retinal layer analysis for other ocular diseases related to ONH. • The data provides Optical coherence tomography (OCT) as well as fundus images for each subject which will help in automated correlation of finding from both image modalities.

## 1 Data description

Fundus Images has been widely used for the initial examination of ophthalmic abnormalities. Ophthalmologist recommends appropriate treatment by observing subtle changes in ONH through fundus images [4]. OCT is relatively fastest imaging technique that provides the quantitative assessment of retinal layers [5]. OCT images are used to observe the morphological changes in retinal layers which provide the detail picture of ocular disease.

The data set include both controlled and glaucomatous case images of Fundus and OCT Images of humans. The data encompass is acquired from 26 subjects scanned on TOPCON'S 3D OCT system. It includes both eye data for 23 subjects and one eye data for three subjects. So, the presented data is comprised of 50 OCT and Fundus images, including 18 controlled and 32 glaucomatous affected cases. The subjects are selected in such a way that they form a diverse dataset as it includes male and female subjects belonging to different age groups. Local ethical committee approved data collection procedure and hospital ethical board approved data collection after getting consent from subjects and proper anonymization of data.

**Fig. 1:**
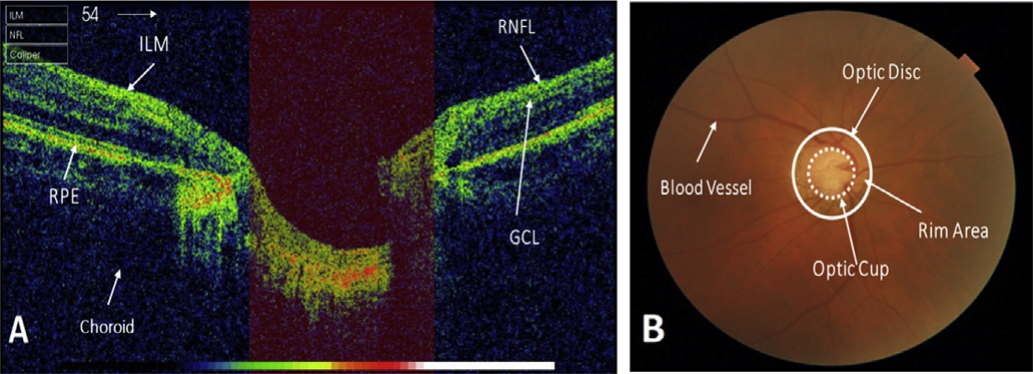
Images of left Eye of a subject. A) An OCT Image showing the retinal layers that are used for Glaucoma diagnosis: Inner ILM layer, RNFL layer, Ganglion cell layer and RPE layer. B) Fundus Image showing optic disc, optic cup, rim area and blood vessels.

**Fig. 2:**
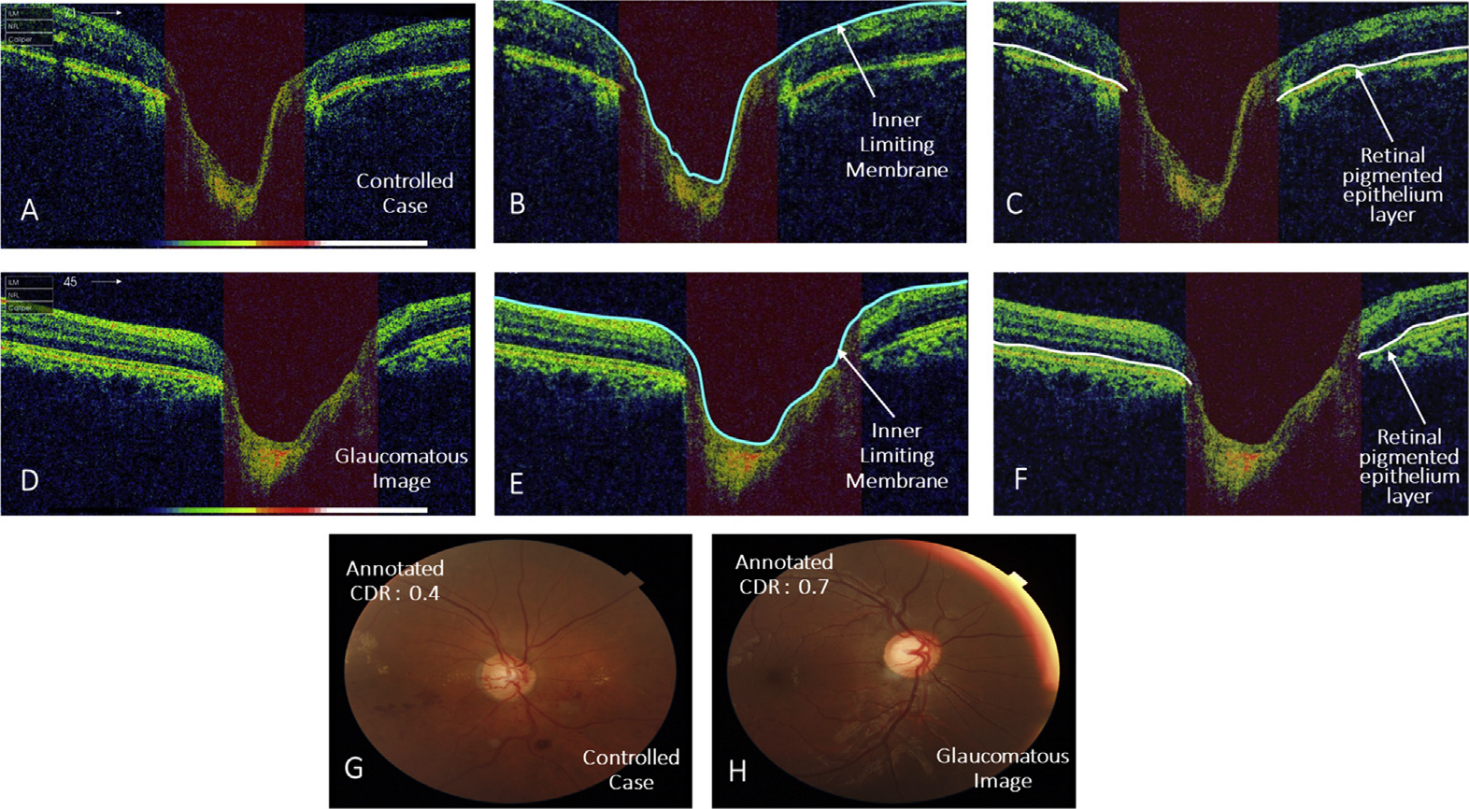
OCT and Fundus Images of controlled and glaucomatous subjects; OCT and Fundus of controlled subject are shown in (A) & (G). The corresponding ILM and RPE layer delineation are shown in (B) & (C). Glaucomatous OCT and Fundus Images are shown in (D) & (H) and manually extracted ILM and RPE layers are highlighted in (E) & (F).

The provided OCT Images in data set are B-scan and ONH centred with resolution of 951 × 456 [6]. Fig. 1 shows the fundus and its corresponding OCT image of a subject from the data set. OCT image is ONH centred; the retinal layers mostly considered for the detection of glaucoma are highlighted in Fig. 1(A). Whereas, Fig. 1(B) shows the fundus image of the same subject that helps in viewing the ONH abnormalities. As OCT images undergoes pre-processing steps for further analysis, so images are cropped and resized to original image 951 × 456. Manually annotation is performed on rescaled image. Manually delineation of the Inner Limiting Membrane (ILM) Layer and Retinal pigmented epithelium (RPE) layer is achieved using Illustrator CS6 with the help of ophthalmologist. The manually outlined ILM and RPE layers through OCT Images of controlled and glaucomatous subject are shown in Fig. 2(B, E) and Fig. 2(C, F) respectively. For each OCT image we provided its Fundus image along with Cup to disc ratio (CDR) annotations performed by four glaucoma specialists. In addition, glaucoma specialist classifies the images into controlled, suspect and glaucoma through analysis of fundus and OCT images. The CDR annotation and labels for each image are provided in excel file. The fundus and OCT images for controlled and glaucomatous subjects are shown in Fig. 2(A, B, C, G) and Fig. 2(D, E, F, H) respectively. It is evident from Fig. 2 the cup size increased in glaucoma case thus as result CDR value also raised.

## 2 Experimental design, materials, and methods

The OCT machine TOPCON'S 3D OCT-1000 system is employed to acquire the OCT and fundus Images. Examination and image acquisition are performed after pupil dilations with Ø4.0mm (45°) diameter. B-scan acquisition frequency is 5Hz thus reducing the eye movement effects. Scanning Speed is 27,000 to 50,000 A-scans per second with the depth of 2.3mm, B-scan is comprised of 1024 A-scans. The Lateral and Vertical resolution are kept 5.9mm (±0.2) and 5.9mm (±0.2) respectively. The OCT scans of retina are optic nerve head (ONH) centred with 951 × 456 resolution.

The data set had been used for the evaluation of automated segmentation algorithm for the extraction of retinal layers [[1], [2], [3]].
